# A comparative study of the feet of middle-aged women in Korea and the Maasai tribe

**DOI:** 10.1186/s13047-015-0126-1

**Published:** 2015-12-01

**Authors:** Jun Young Choi, Sang Hyun Woo, Sang Hyun Oh, Jin Soo Suh

**Affiliations:** W Institute for Foot and Ankle Disease & Trauma, W Hospital, 101-6, Gamsam-dong, Dalseo-gu, Daegu South Korea; Department of Orthopedic Surgery, Inje University Ilsan Paik Hospital, 170 Juhwa-ro, Ilsanseo-gu, Goyang-si, Gyeonggi-do South Korea

**Keywords:** Maasai, Maasai foot, Barefoot, Unshod foot

## Abstract

**Background:**

Members of the Maasai tribe spend their days either barefoot or wearing traditional shoes made from recycled car tires. Although they walk long distances (up to 60 km) daily, they do not generally experience foot ailments. Here, we compared parameters associated with the feet, ankles, and gait of middle-aged women in Korea and the Maasai tribe.

**Methods:**

Foot length, calf circumference, hindfoot alignment, step length, cadence, and walking velocity were compared among 20 middle-aged Korean and bush-living Maasai women. Static and dynamic Harris mat footprints were taken to determine the distribution of forefoot pressure patterns during walking. We also compared several radiographic parameters with standing foot and ankle radiographs.

**Results:**

The mean foot length and width were significantly longer in Maasai women. Interestingly, 38 ft (96 %) in the Maasai group showed a claw deformity of the toe (s). There were no statistically significant differences in gait-related indices and Harris mat findings between the two groups. On radiographic analysis, tibial anterior surface angle, tibial lateral surface angle, talonavicular coverage angle, talo-first metatarsal angle, Meary angle, and naviculo-cuboidal overlap were significantly greater in the Maasai group, whereas hallux valgus angle and the first and second intermetatarsal angle were greater in the Korean group.

**Conclusions:**

Middle-aged women from the Maasai tribe showed a higher prevalence of abducted forefeet, everted hindfeet, and fallen medial longitudinal arches than did Korean women, while Korean women showed a higher prevalence of hallux valgus, a preserved medial longitudinal arch, and toes that are free from claw deformity.

## Background

In modern society, most people would not consider walking without shoes for even 1 day. However, we know that our ancestors lived barefoot or in lighter shoes than those worn today. Shoes protect the feet from harmful objects on the ground while an individual is walking and running. However, shoes also cause many kinds of foot problems. Kadambande et al. [1] mentioned that shod feet appeared to have a significant reduction in pliability compared to the unshod foot. Sim-Fook and Hodgson [2] reported that the foot in its natural unrestricted form was mobile and flexible without any of the static complaints often encountered from individuals wearing shoes.

The Maasai are the most widely known indigenous ethnic group in Africa. Currently, the Maasai population is estimated to be 883,000, with the majority residing widely in southern Kenya and northern Tanzania [3]. Because of their semi-nomadic lifestyle, most of the Maasai still spend their days either barefoot or wearing a pair of traditional shoes made from recycled car tires. Although they walk long distances (up to 60 km) daily, they do not generally experience foot ailments such as frequent sprain, persistent pain, and osteoarthritis of the midfoot and ankle joint. Our previous study reported the salient features of the feet of 1096 Maasai people grouped by age and sex [4]. We found that 5.84 and 1.92 % of Maasai participants had bilateral and unilateral fallen medial longitudinal arches (<1 cm), respectively. Overall, 98.79 % of Maasai participants had a claw deformity of at least one toe. Regarding our previous study, we hypothesized that women from the Maasai tribe, who spend most of the day partially shod, would be more likely to have reduced medial longitudinal arches and claw toe deformities. A comparative study including radiographic parameters related to the gait and feet of middle-aged Maasai women living partially shod in the bush to those of middle-aged women living in modern shoe-wearing society was performed. We assume that the feet of middle-aged women would reflect their lifestyle rather than the aging process. Incidence of foot diseases affected by shoes is high in middle aged woman. Coughlin and Jones [5] stated that 65 % of adults reported the onset of their hallux valgus deformity in the third through fifth decades. Coughlin and Thompson [6] reviewed more than 800 cases of hallux valgus and reported the mean age at surgery to be 60 years. With respect to degenerative arthritis of the ankle joint, autopsy studies demonstrated that the prevalence of advanced degenerative changes increased with age [7, 8].

To our knowledge, the present study is the first to compare parameters associated with the feet, ankles, and gait of middle-aged Korean and Maasai women directly.

## Methods

### Participants

A total of 20 healthy Maasai women aged 46–55 years from various rural villages around Arusha, Tanzania, volunteered for this observational study from September 2012 to March 2013. Initially, women who had no neuromuscular dysfunction while walking a long distance as part of their daily lives were chosen for the study. From those, patients who denied a history of medical illness (e.g.,rheumatic arthritis, gout), trauma, or symptoms that could be related to foot problems were ultimately included in the study. Exclusion criteria were the presence of any kind of lower extremity length discrepancy, excessive angular deformation, or limping gait. A similar study with the same number of Korean women under the same conditions who volunteered for this study from among hospital visitors was carried out in Seoul from June 2014 to December 2014. In that study, height and weight were measured and body mass index (BMI) was calculated. The present study compared data from the two groups of women. Informed consent was obtained from all participants, and the studies were approved by the ethical review committee of our institution.

### Measuring surface anatomy

The length of both feet were measured from the most posterior part of the heel to the most anterior part of the longest toe while in a full weight-bearing position. The width of both feet, defined as the distance between the most medial part of the forefoot and the point that meets the perpendicular line to the heel bisecting line from the most medial part of the forefoot, was measured [9]. The ratio of foot width to length was calculated. At the same time, the calf circumference was measured at the widest point of the calf using a flexible measuring tape. All measurements were taken by a single, trained person to minimize inter-and intra-observer errors.

Static measurements of hindfoot alignment involved goniometric measurements of the motion between the calcaneus and lower third of the leg in the frontal plane. Each participant was positioned with her feet flat in a standing position with the clinician standing behind the patient. The clinician then positioned the participant in a subtalar neutral position, which was determined by palpating the talar head and positioning it to be congruent to the navicular bone. The angle between the bisecting lines of the calcaneus and lower third of the leg was measured with a protractor [10].

The height of the arch was defined as the highest point on the navicular tuberosity of the medial longitudinal arch of the foot [11]. Measurements were taken while the right foot was in a full weight-bearing position with the left foot resting lightly on the floor. The ankle joint was placed in a neutral position with the body in a normal upright posture [12]. A series of measurements was taken to determine the highest point along the navicular tuberosity of the medial plantar curvature of the foot.

All feet were examined by a single, trained clinician to detect any toe deformities, such as a mallet or claw toe, and accompanying soft or hard corn formation. Mallet toe deformity was defined as a flexion of the distal interphalangeal (DIP) joint accompanied by hyperextension of the proximal interphalangeal (PIP) joint, whereas claw toe deformity was defined as hyperextension of the metatarsophalangeal joint with flexion of the PIP and DIP joints.

### Measuring step length, cadence, and walking velocity

To measure the step length, cadence, and walking velocity, each participant walked barefoot on a flat surface for 1 min. During their walk, the number of steps per minute (cadence) was counted and the distance walked in a given time (walking velocity) was measured. Step length was measured from the heel of a footprint of one foot to the heel print of the other foot or the distance traveled forward by a single leg (stride).

### Harris and Beathfootprinting

**Fig. 1:**
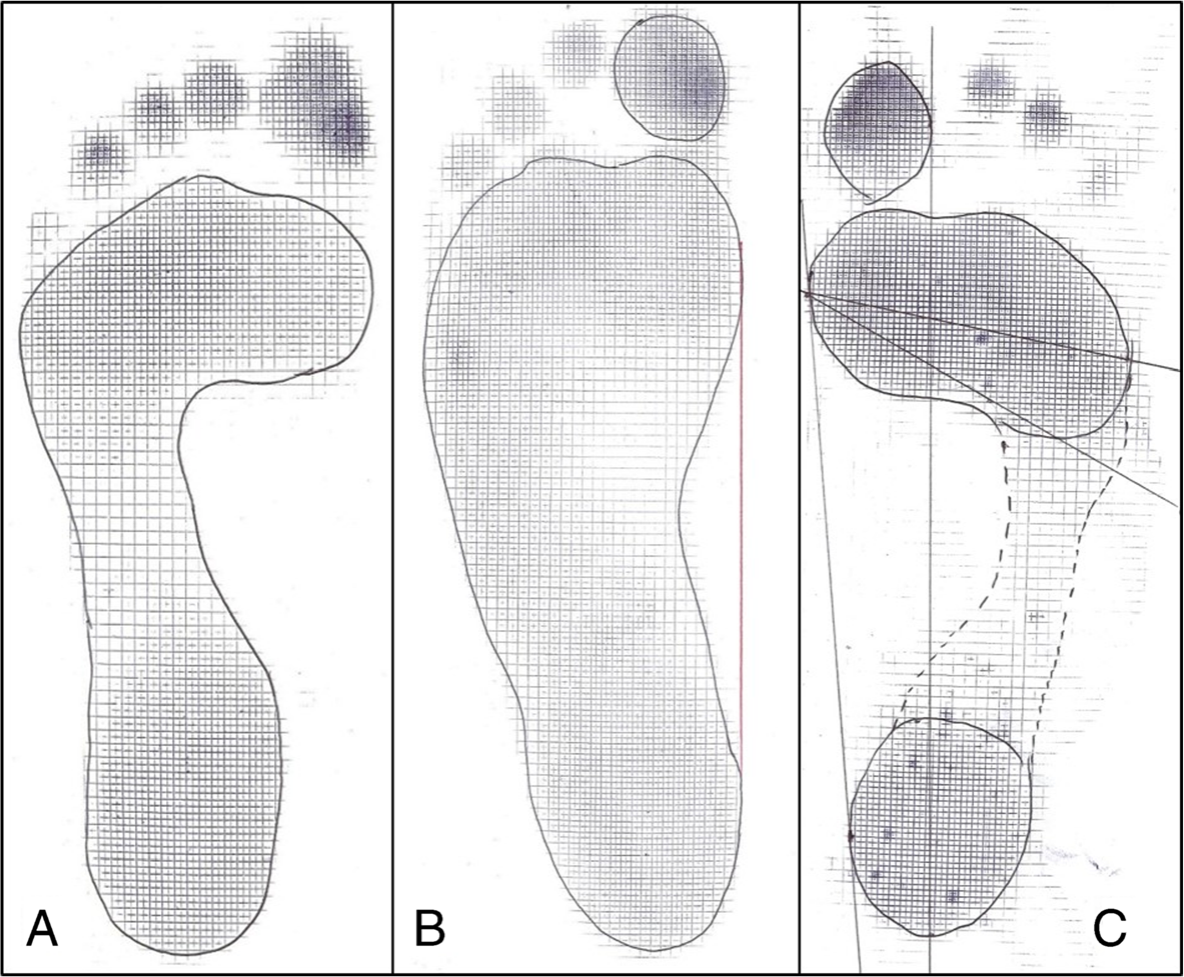
Normal (**a**), broad (**b**), and high (**c**) arched footprint patterns

Standing (static) footprints were made with the participant looking ahead with her hands by her side and the feet comfortably together with a small gap between the medial malleoli. When a walking (dynamic) footprint was taken, the participant was asked to walk normally for a few steps on the smooth surface of the mat. Several prints of each foot were made until the investigator was satisfied by the reproducibility and confident that the dynamic and static characteristics were truly represented. Footprints were subjectively analyzed for dynamic heel shape and pressure, direction of the heel bisecting line, and dynamic forefoot pressure by a single observer and matched with the clinical examination findings of the static and dynamic foot shape to determine whether the footprint had a broad footprint pattern or a high-arched pattern (Fig. 1) [13].

### Radiological evaluation

Radiographs in the anteroposterior (AP) view of the weight-bearing foot, AP view of the ankle, and lateral view of the foot and ankle joint were obtained to evaluate several parameters related to foot and ankle deformities. On AP weight-bearing ankle radiographs, the tibial anterior surface angle (TAS) [14] and talar tilt were measured to evaluate the alignment of the ankle joint. The hallux valgus angle (HVA) [15] and the first and second intermetatarsal angles (IMAs) were measured to determine the tendency for the hallux valgus to develop after wearing shoes for a lifetime. HVA was defined as the angle between the axes drawn on the first metatarsal and the proximal phalanx, and the IMA was measured by drawing reference points in the proximal and distal metaphyseal regions equidistant from the medial and lateral cortices of the first and second metatarsals. The talonavicular coverage angle (TNCA) [16] and talo-first metatarsal angle (T1MTA) [17] were also measured to evaluate the degree of forefoot abduction/adduction on the weight-bearing foot on AP radiographs. TNCA was the angle between a line bisecting the anterior articular surface of the talus and another line bisecting the proximal articular surface of the navicular bone. T1MTA was defined as the angle between a line bisecting the anterior articular surface of the talus and a line bisecting the long axis of the first metatarsal. On the lateral weight-bearing radiographs, the talo-first metatarsal angle (Meary angle) [17] and the calcaneal pitch angle (CPA) [18] were measured to evaluate the tendency of having pes planus/cavus and hindfoot eversion/inversion. The Meary angle was the angle between a line bisecting the long axis of the first metatarsal bone and a line drawn through the midpoints of the talar head and neck. CPA was the angle between a line drawn along the edge of the plantar soft tissue shadow and a line drawn along the lower margin of the calcaneus. In addition, the naviculo-cuboid overlap (NCO) [19] was measured on lateral weight-bearing radiographs to evaluate the degree of hindfoot eversion or inversion. NCO was defined as the portion of the navicular bone divided by the vertical height of the cuboid.

Measurements on the radiographs were taken manually on traditional X-ray films in Tanzania and digitally with a computer program in Korea.

### Statistical analysis

The mean values and standard deviations for all dependent parameters were calculated using SPSS 18. The Wilcoxon signed-rank test was used to compare differences in radiographic measurements between the two groups. A *p*-value of <0.05 was considered significant for all analyses.

## Results

### Participant demographics

The mean age of the Korean and Maasai groups were 49.1 ± 3.28 years and 48.55 ± 3.76 years, respectively. The demographic parameters of each group are summarized in Table 1. There were no statistically significant differences in demographic parameters.

**Table 1:** Participant demographic characteristics

	Korean women	Maasai women	*p*-value
Age (years)	49.1 ± 3.28	48.55 ± 3.76	0.727
Body weight (kg)	62.0 ± 8.91	57.3 ± 12.03	0.219
Body height (cm)	160.7 ± 3.91	159.55 ± 5.60	0.283
BMI (kg/m^2^)	24.06 ± 3.72	22.44 ± 4.30	0.24

*BMI*, body mass index

### Surface anatomy (Table 2)

**Table 2:** Surface anatomy of each group

	Korean women	Maasai women	*p*-value
Foot length (cm)	234.75 ± 6.78	243.5 ± 12.15	0.005
Foot width (cm)	95.59 ± 4.53	99.73 ± 5.02	0.001
Foot width/ft length	0.41 ± 0.02	0.41 ± 0.01	0.586
Calf circumference (cm)	35.13 ± 3.03	34.05 ± 3.24	0.355
Hindfoot alignment (°)	5.97 ± 3.36 (eversion)	7.1 ± 2.07 (eversion)	0.098
Arch height (cm)	1.58 ± 0.73	1.55 ± 1.37	0.646
Number of flat feet (of 40)	0 (0 %)	4 (10 %)	
Number of claw toes (of 40)	0 (0 %)	38 (95 %)	

The mean foot length and width were significantly longer in Maasai women, but the ratio of foot width to length was not significantly different between the two groups. In the Maasai group, four participants had an excessively low arch (<0.5 cm), which is defined as a flat foot. Interestingly, 38 ft (19 pairs) (96 %) in the Maasai group showed claw deformity of the toes. The number of the affected toe varied, with the highest incidence seen on the fifth toe (38 of 38 ft, 100 %). The most common hindfoot alignment was valgus in both groups, which was pronounced in the Maasai group but not statistically significantly so. The exception was a case of hindfoot varus, which was observed only in the Korean group.

### Step length, cadence, and walking velocity (Table 3)

**Table 3:** Parameters related to gait

	Korean women	Maasai women	*p*-value
Step length (cm)	36.83 ± 11.46	43.35 ± 6.94	0.01
Cadence	114.8 ± 12.45	95.1 ± 3.39	0.001
Walking velocity (m/min)	42.81 ± 15.16	41.17 ± 6.48	0.681

Step length was significantly longer in the Maasai group, while the cadence was higher in the Korean group. However, the walking velocity was not statistically different between the two groups.

### Footprint analyses (Table 4)

**Table 4:** Footprint analysis

		Korean women (*n* = 40)	Maasai women (*n* = 40)
Heel shape		All oval shape	All oval shape
Heel pressure		All even pressure	All even pressure
Footprint shape (High arch/normal/broad)		All normal	Broad type in 4 cases
Heel bisecting line	1^st^web space	10 (25 %)	2 (5 %)
	2^nd^ toe	9 (22.5 %)	14 (35 %)
	2^nd^web space	20 (50 %)	24 (60 %)
	3^rd^ toe	1 (2.5 %)	0 (0 %)
Forefoot pressure	Medial	3 (7.5 %)	4 (10 %)
	Middle	1 (2.5 %)	0 (0 %)
	Lateral	10 (25 %)	9 (22.5 %)
	Even	26 (65 %)	27 (67.5 %)

**Fig. 2:**
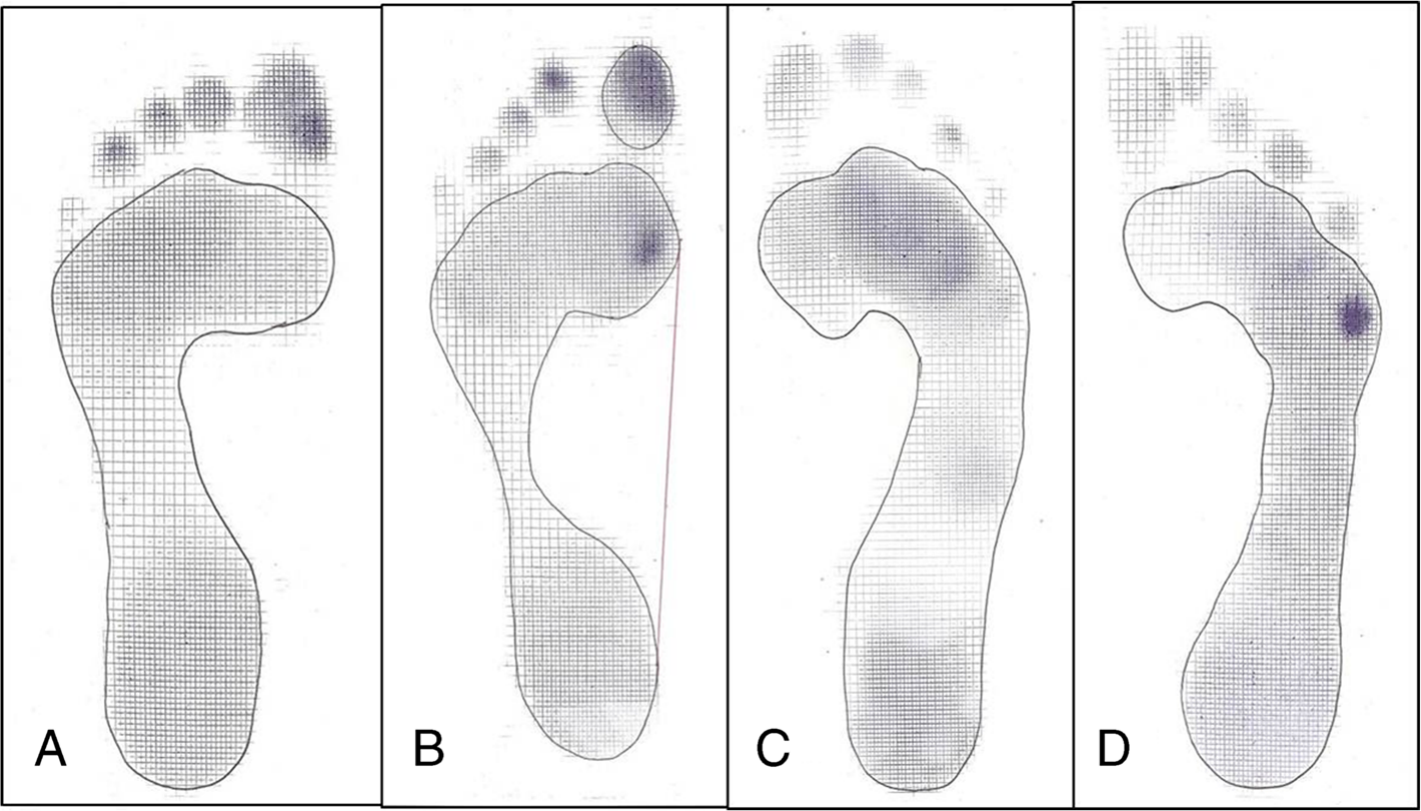
Even (**a**), medial (**b**), middle (**c**), and lateral (**d**) dynamic forefoot pressure patterns

All participants had an oval-shaped, even-pressured heel with the bisecting line directed between the first and third toes. In the Maasai group, four participants had a broad footprint pattern with static footprints, which perfectly matched the definition of flat foot deformity. For forefoot pressure with dynamic footprints, the participants were divided into four different groups as follows: (1) even pressure throughout the forefoot, (2) medial pressure under the first metatarsal head, (3) middle-pressure under the second to third metatarsal heads, and (4) lateral pressure under the fifth metatarsal head spreading to include the fourth metatarsal head (Fig. 2). In the Korean and Maasai groups, the even-pressure concentration pattern was most frequent, followed by lateral and medial.

### Radiological evaluation (Table 5)

**Table 5:** Radiologic parameters of the two groups

		Korean women	Maasai women	*p*-value
Weight-bearing ankle AP & lateral	TAS (^o^)	88.85 ± 2.21	91.67 ± 1.65	0.0001
	TLS (^o^)	79.7 ± 2.27	83.97 ± 1.96	0.0005
	Talar tilt (^o^)	1.47 ± 1.06	1.58 ± 0.63	0.238
Weight-bearing foot AP	TNCA (^o^)	15.60 ± 7.06	31.95 ± 10.23	0.001
	T1MTA (^o^)	11.70 ± 6.25	26.53 ± 5.42	0.0005
	HVA (^o^)	13.99 ± 4.88	7.57 ± 4.43	0.001
	IMA (^o^)	10.86 ± 2.19	7.59 ± 2.17	0.005
Weight-bearing foot lateral	Meary angle (^o^)	9.08 ± 7.08	15.55 ± 6.46	0.005
	CPA (^o^)	17.19 ± 5.31	15.98 ± 3.81	0.371
	NCO (ratio)	0.44 ± 0.14	0.54 ± 0.12	0.001

*TAS* talar anterior surface angle, *TLS* talar lateral surface angle, *TNCA* talo-navicular coverage angle, *T1MTA* talo-1st metatarsal angle, *HVA* hallux valgus angle, *IMA* intermetatarsal angle, *CPA* calcaneal pitch angle, *NCO* naviculo-cuboid overlap

On the AP and lateral images of the weight-bearing ankle, TAS and TLS were significantly greater in the Maasai group. Talar tilt was not significantly different between the two groups.

**Fig. 3:**
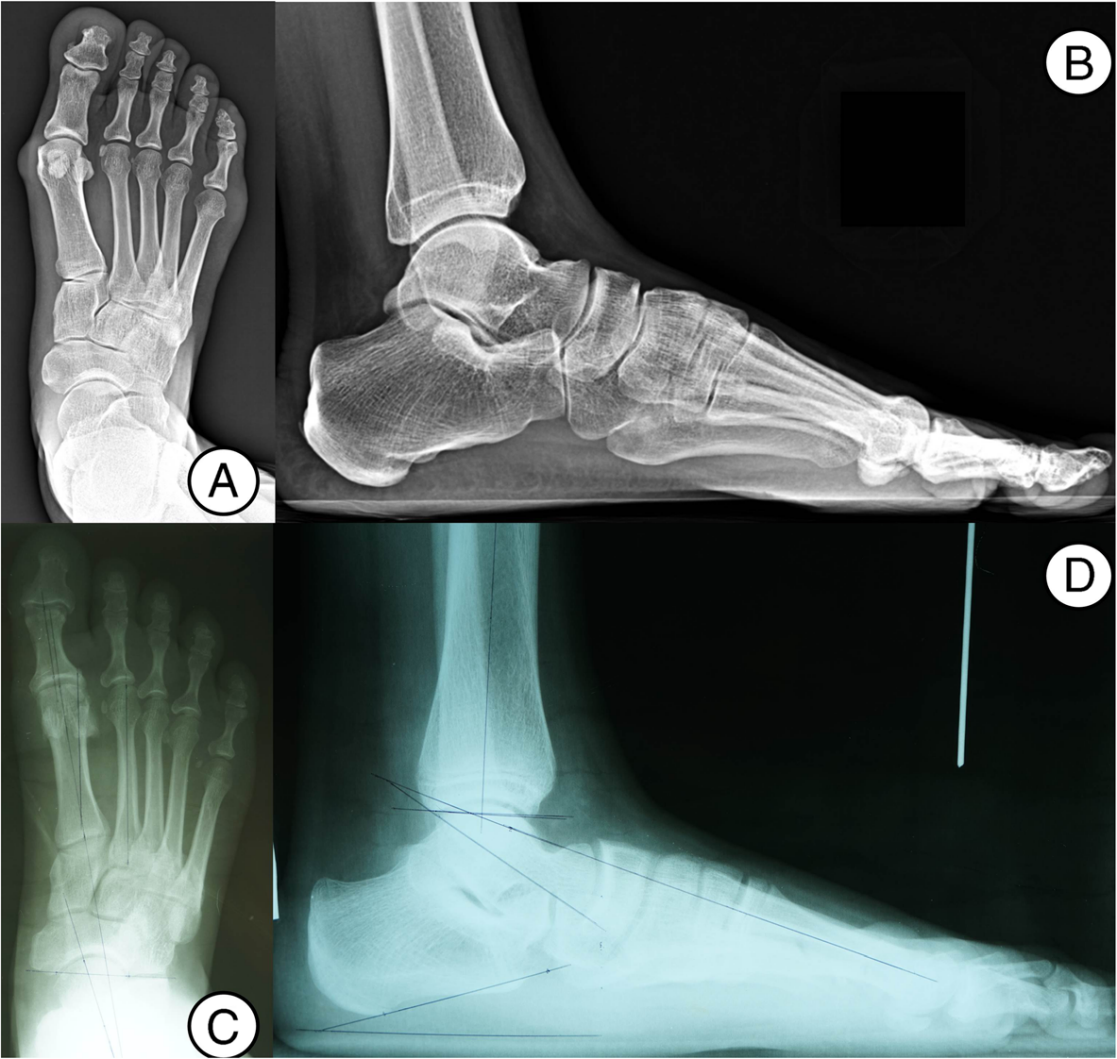
Foot AP and lateral radiographs with standing (**a**, **b** Maasai, **c**, **d** Korean). The TNCA and T1MTA were significantly greater in the Maasai group on the AP images of the weight-bearing foot, indicating more forefoot abduction related to hindfoot eversion. HVA and IMA were significantly greater in the Korean group. On the lateral images of the weight-bearing foot, the Meary angle and NCO were significantly increased in the Maasai group

The TNCA and T1MTA were significantly greater in the Maasai group on the AP images of the weight-bearing foot, indicating more forefoot abduction related to hindfoot eversion. HVA and IMA were significantly greater in the Korean group. On the lateral images of the weight-bearing foot, the Meary angle and NCO were significantly increased in the Maasai group (Fig. 3).

## Discussion

To our knowledge, this is the first study comparing the foot shape and gait of both Korean and Maasai women. A previous study revealed radiological differences based on ethnicity between African Americans, Caucasians, and Hispanics and indicated that African Americans had a significantly lower CPA compared to that in the other ethnic groups [20]. However, in contrast to our study, all the participants in that study had been born and raised in a developed society. We used data from a single, specific, independent group from Korea and from women of the Maasai tribe, who dwell partially shod. Thompson and Zipfel [9] compared the forefoot morphology of two different populations who had similar histories of partially shod behavior in childhood. They found that there were no appreciable differences in the distribution of forefoot width ratios between adult Caucasian and African populations in South Africa. In our study, the foot length and width were shown to be significantly larger in the Maasai group, whereas the ratio of foot width to length was the same in the two groups. An everted position in the hindfoot was seen more frequently in the Maasai group than in the Korean group; however, this was not statistically significant. The mean arch height was not significantly different between the two groups.

**Fig. 4:**
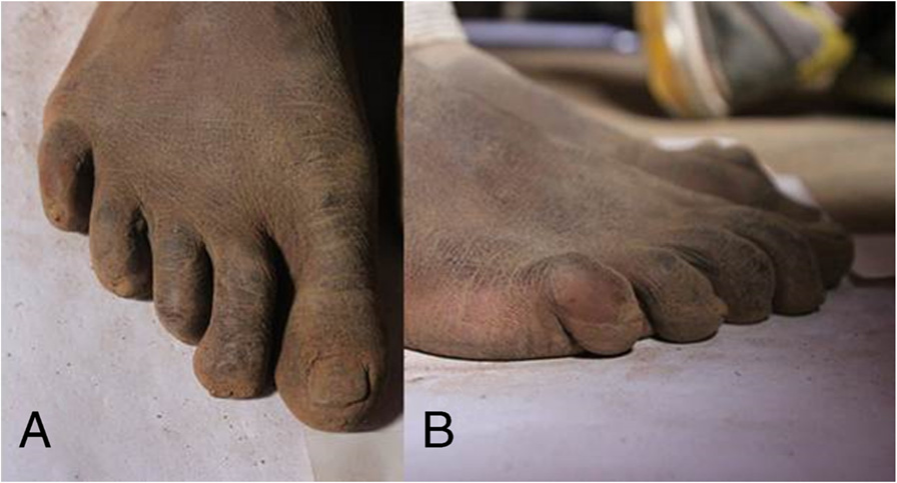
Claw toe deformity in the Maasai group (**a**). Hyperextension of the metatarsophalangeal joint and flexion of the proximal interphalangeal and distal interphalangeal joints are shown (**b**)

Interestingly, the prevalence of claw toe deformity (Fig. 4) was significantly higher in the Maasai group than in the Korean group (95 % vs. 0 %), which is in agreement with results from a previous study [4]. In that previous study, claw toe deformity occurred in 98.79 % of 1096 Maasai people, with the fifth (62.34 %) being the most frequently affected toe. It is highly plausible that living barefoot in the bush could contribute to the occurrence of claw toe deformity. Further studies are needed to clarify this finding.

In terms of gait parameters, Maasai women walked slower but farther in a single step compared to how their Korean counterparts walked. Therefore, the walking velocity was not significantly different between the groups.

There was no significant difference found in the Harris and Beath footprints. The shape and pressure of the heels were noted to be oval and uniform in all participants. A heel bisecting line is a line drawn through the midline axis of the hindfoot and forefoot. This line has been shown to be helpful for evaluating forefoot adduction and abduction. In a neutral foot, the heel bisecting line passes through the second web space. In our study, the line passed through the second web space in the majority of participants, followed by the second toe and the first space. Over 65 % of the participants in both groups showed uniform forefoot pressure concentrations with dynamic footprints, followed by lateral pressure concentration, which indicates an over-supinated forefoot position.

On radiographic examination, we measured the parameter related to hindfoot inversion or eversion on the basis of a study by Lee et al. [21] that reported that NCO, TNCA, and T1MTA were reliable and valid measures for the evaluation of hindfoot eversion and inversion deformities. In our study, the NCO, TNCA, and T1MTA were significantly higher in the Maasai group.

It is well recognized that hallux valgus occurs almost exclusively in people who wear shoes in their daily activities compared to those who occasionally wear shoes [22].

Trinkaus [23] mentioned that hallucal robusticity could be significantly affected by the use of footwear in an archaeological study. Shine [24] reported that hallux valgus was found in less than 2 % of the unshod and in 16 % of men and 48 % of women who had worn shoes for more than 60 years. Zipfel and Berger [25] also suggested that the pathological variation in the metatarsus was affected by habitual behavior, including the wearing of footwear and exposure to modern substrates. Our results reveal findings similar to those in a previous report [26], which showed that HVA and IMA were substantially increased in the Korean group who spent their entire lives in ready-made shoes. The TAS was also found to be significantly higher in the Maasai group, and this result was similar to that in a previous study performed in the United States [27]. The TAS of the Korean group in our study was similar to that in previous reports from Korea [28] and Japan [14]. In our study, however, the TLS in the Maasai group was shown to be significantly higher than TLS results that have previously been reported [28].

A limitation of this study is that we could not collect and compare data from either Maasai who wear shoes regularly or from Koreans who do not wear shoes regularly. Genetic and ethnic factors might have played independent roles in the outcomes of our study. In order to obtain a more comprehensive dataset, further studies should investigate and compare similar data from young participants. Another limitation of the study is the small number of participants in each group.

## Conclusions

In conclusion, we report that middle-aged women from the Maasai tribe showed more abducted forefeet, everted hindfeet, and fallen medial longitudinal arches than did Korean women, while Korean women were more likely to have hallux valgus, preserved medial longitudinal arches, and toes free from claw deformity. As these results could have been affected by either living partially shod in the bush or living in a modernized society where people wear ready-made shoes, further comparative studies are necessary. Even though the total number of the participants was small, we believe our results suggest different interesting parameters between Korean women and women from the Maasai tribe.
